# How much information can be obtained from tracking the position of the leading edge in a scratch assay?

**DOI:** 10.1098/rsif.2014.0325

**Published:** 2014-08-06

**Authors:** Stuart T. Johnston, Matthew J. Simpson, D. L. Sean McElwain

**Affiliations:** 1 Mathematical Sciences, Queensland University of Technology, Brisbane, Australia; 2 Institute of Health and Biomedical Innovation, Queensland University of Technology, Brisbane, Australia

**Keywords:** cell motility, cell proliferation, scratch assay, edge detection, cancer

## Abstract

Moving cell fronts are an essential feature of wound healing, development and disease. The rate at which a cell front moves is driven, in part, by the cell motility, quantified in terms of the cell diffusivity *D*, and the cell proliferation rate *λ*. Scratch assays are a commonly reported procedure used to investigate the motion of cell fronts where an initial cell monolayer is scratched, and the motion of the front is monitored over a short period of time, often less than 24 h. The simplest way of quantifying a scratch assay is to monitor the progression of the leading edge. Use of leading edge data is very convenient because, unlike other methods, it is non-destructive and does not require labelling, tracking or counting individual cells among the population. In this work, we study short-time leading edge data in a scratch assay using a discrete mathematical model and automated image analysis with the aim of investigating whether such data allow us to reliably identify *D* and *λ*. Using a naive calibration approach where we simply scan the relevant region of the (*D*, *λ*) parameter space, we show that there are many choices of *D* and *λ* for which our model produces indistinguishable short-time leading edge data. Therefore, without due care, it is impossible to estimate *D* and *λ* from this kind of data. To address this, we present a modified approach accounting for the fact that cell motility occurs over a much shorter time scale than proliferation. Using this information, we divide the duration of the experiment into two periods, and we estimate *D* using data from the first period, whereas we estimate *λ* using data from the second period. We confirm the accuracy of our approach using *in silico* data and a new set of *in vitro* data, which shows that our method recovers estimates of *D* and *λ* that are consistent with previously reported values except that that our approach is fast, inexpensive, non-destructive and avoids the need for cell labelling and cell counting.

## 1. Introduction

Moving cell fronts are key features of tissue repair [1] and tumour spreading [2]. The rate at which the front of a population of cells moves is influenced by the rate at which individual cells within the population migrate and proliferate [3]. Random, undirected cell migration is typically quantified in terms of the cell diffusivity *D*, whereas cell proliferation is quantified in terms of the proliferation rate *λ*. Developing methods to estimate *D* and *λ* from experimental observations is important so that we can assess the effectiveness of intervention strategies which often aim at influencing either *D* or *λ* [4,5]. For example, drugs such as mitomycin-C, which inhibit proliferation [4], are used to reduce tumour spreading [5], whereas steroid treatment, which stimulates cell migration [6], is often studied with the aim of enhancing wound healing.

Scratch assays [7–10], also known as scrape or wound-healing assays [9,10], are routinely used to investigate the motion of cell fronts by creating a scratch in a cell monolayer and observing the motion of the cell front. Images of the front are captured over a period of time that is typically less than 24 h [7,11,12]. Use of short-time-scale experimental data is very common because it avoids the need for replenishing the nutrients in the assay. There are various ways that data from a scratch assay are reported and analysed. The most common method is to present a qualitative, visual comparison between a control assay and another assay where some treatment has been applied. This kind of data is often presented without any attempt to estimate *D* or *λ*. For example, Teppo *et al*. [9] presented scratch assay data showing that hypoxia increased the rate at which the fronts of cancer cells moved, but they did not determine how the hypoxic conditions affected *D* and/or *λ*.

Another approach to analyse scratch assays is to use a mathematical model, such as the Fisher–Kolmogorov equation [13] or an extension of this reaction–diffusion equation [14–17] (electronic supplementary material). Some previous studies have focused on matching the experimental front speed with the long-time asymptotic travelling wave speed of the Fisher–Kolmogorov equation, 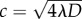 [18–20]. Unfortunately, this approach is of little practical use for most experiments which are conducted over short time scales where no such travelling wave forms [7,11,12]. Another way of analysing scratch assays is to generate cell density profiles which can be matched to numerical solutions of a reaction–diffusion equation [3,21,22]. Unfortunately, this approach is expensive and time consuming because it requires some kind of direct or indirect cell counting technique to construct the density profiles. Other mathematical models have been used to interpret scratch assays, such as mechanistic [23] and biased continuum models [24]. However, the experimental procedures required to parametrize these models are time consuming because they involved individual cell counting [23] or individual cell tracking [24].

The simplest and most cost-effective measurement that can be made to characterize a scratch assay is to record the location of the cell front as a function of time [8,11,25]. The widespread availability of automatic edge detection algorithms [26,27] means that it is straightforward to obtain this information. Given that most scratch assays are conducted for short time periods, here we seek to determine whether it is possible to reliably estimate *D* and *λ* from short-time leading edge data alone without constructing cell density profiles [3,21,22]. To explore this question, we use automatic edge detection algorithms to analyse a discrete model of collective cell spreading driven by cell migration and cell proliferation [28]. While such models have been used to analyse various types of *in vitro* assays previously [21,29], these studies have not focused on short-time leading edge data. Our work shows that great care must be taken when interpreting short-time leading edge data because the most straightforward model calibration approach indicates that there are many choices of *D* and *λ* which lead to indistinguishable leading edge data. To overcome this, we develop a novel method by dividing the leading edge time-series data into two intervals allowing us to estimate *D* from the first time interval, and then we separately estimate *λ* using the second time interval. We test the method using both *in silico* and *in vitro* data showing that we recover estimates of *D* and *λ* that are consistent with previously reported results obtained using far more complicated experimental procedures.

This article is organized in the following way. In §2, we describe a discrete model for simulating the motion of cell fronts. Section 2 describes the image analysis and experimental procedure. Data in §3 show that a straightforward model calibration procedure implies that there are many choices of *D* and *λ* that match short-time leading edge data. As a result, we also describe, in §3, a modified method that leads to unique estimates of *D* and *λ*, and we validate our results using both *in silico* and *in vitro* data. Finally, in §4, we discuss our results and outline options for extending the work.

## 2. Material and methods

### 2.1. Experimental method

**Figure 1.**
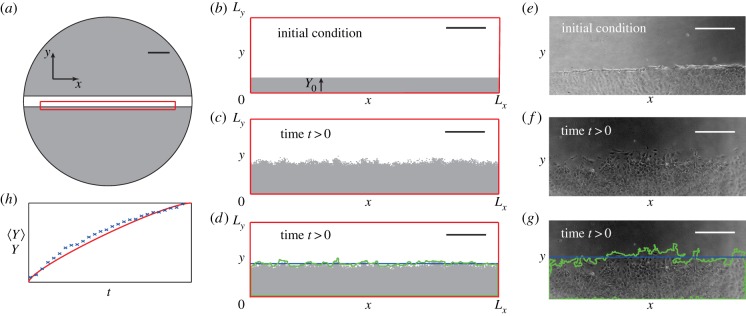
Schematic illustrating the initial scratch in the cell monolayer, simulation and leading edge data. (*a*) Monolayer of cells (grey) immediately after the scratch (white) has been made. The red rectangle indicates the spatial region which we simulate. (*b*) The initial confluent cell monolayer (grey) has height *Y*_0_ and the width *L_x_*, corresponding to the width of the red rectangle in (*a*). The height of the domain *L_y_* is chosen to be sufficiently large that the agents in the simulation never touch this boundary within the 24 h period of the simulation. (*c*) Simulation after time *t*. (*d*) The simulation results are analysed using the image analysis tools to detect the leading edge (green) which is used to estimate the average position of the leading edge (blue). Scale bars in (*a*–*d*) are 2 mm. (*e*) Typical experimental image immediately after a scratch has been made. To illustrate the edge detection algorithm, we show in (*f*) an experimental image and in (*g*) the detected leading edge (green) and the average position of the front (blue). Scale bars in (*e*–*g*) are 400 µm. (*h*) Typical temporal evolution of the position of the leading edge for experimental data (blue crosses) and averaged simulation data (red).

The experimental method has been presented previously [3]. Briefly, murine fibroblast 3T3 cells [30] were grown in T175 cm^2^ tissue culture flasks and 1 μl of cell suspension was placed into the well, with diameter 15.6 mm, of a tissue culture plate. The tissue culture plate was incubated at 37°C and 5% CO_2_ until the population became confluent. A scratch was made in the monolayer using a P1000 pipette tip (Lab Advantage, Australia). Images were recorded at *t* = 0, 3, 6, 9, 12 and 24 h, and a schematic illustration of the assay at *t* = 0 is given in figure 1*a*.

### 2.2. Mathematical model

We consider a lattice-based random walk model on a two-dimensional square lattice, with lattice spacing Δ [28,31]. Each site may be occupied by, at most, one agent, and each simulation contains a total of *Z*(*t*) agents which have the ability to move and proliferate, with probability 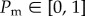 and 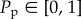, respectively, during each time step of duration *τ*. We make the standard assumption that *P*_m_ and *P*_p_ are constants, which are related to *D* and *λ* by2.1
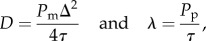
which means that we can view the parameters (*P*_m_, *P*_p_) as being interchangeable with (*D*, *λ*). During each time step *Z*(*t*), agents are chosen, at random, one at a time, and given the opportunity to move [28]. An agent at (*x*,*y*) will attempt to step to (*x* ± Δ, *y*) or (*x*, *y* ± Δ), with the target site chosen with equal probability. After *Z*(*t*) potential motility events have been attempted, an additional *Z*(*t*) agents are selected, at random, one at a time, and given the opportunity to proliferate. A proliferative agent at (*x*,*y*) will attempt to place a daughter agent at (*x* ± Δ, *y*) or (*x*, *y* ± Δ), with the target site chosen with equal probability. Potential motility and proliferation events will only succeed if the target site is vacant, otherwise the event is aborted. Implicitly, this means that individual agents in crowded regions will be relatively immobile and unable to proliferate, whereas uncrowded agents will behave differently and will have a greater opportunity to move and proliferate. The continuum-limit description of this model is a generalization of the Fisher–Kolmogorov equation in two dimensions [28] (electronic supplementary material). This description is valid only when the ratio *P*_p_/*P*_m_ is sufficiently small [28,32].

We apply this model to mimic the geometry of the scratch assay. In all results, we set Δ = 25 µm, corresponding to a typical cell diameter [3,33]. The simulation domain, shown in figure 1*b*, is 0 ≤ *x* ≤ *L_x_* and 0 ≤ *y* ≤ *L_y_*. We choose *L_x_* = 12.5 mm so that our domain captures almost the entire population within the well without directly simulating the curved boundaries. Although it is possible to simulate such curved geometries [3,33], we neglect these details here because our experimental data, described in §3.3, focus on several rectangular subregions within the well, away from the circular boundary. We choose *L_y_* = 3.75 mm which is sufficient to ensure that agents in simulations never reach the boundary, *y* = 3.75 mm, during the 24 h simulation period. Symmetry conditions are applied along the lines *x* = 0, *x* = *L_x_*, *y* = 0 and *y* = *L_y_*. To match our experimental conditions, agents are initially placed on the lattice so that the region *y* < *Y*_0_ is confluent. All simulation data are presented for a particular choice of *τ*, and we re-simulated all results with smaller values of *τ* to ensure our results are insensitive to *τ*.

The results in this work could have been generated using a lattice-free model [34,35]. Instead, we chose a lattice-based model because lattice-free models with crowding effects are far more computationally expensive [34,35]. Furthermore, our recent work showed that lattice-based and lattice-free models produce equivalent data at the leading edge [34,35] which means that there is no advantage in using a lattice-free model here if we are focusing on leading edge data.

### 2.3. Image analysis

We use Matlab's Image Processing Toolbox to estimate the position of the leading edge from the experimental and modelling images. The experimental image is imported and converted to greyscale using imread and rgb2gray, respectively. The simulation data are converted from a matrix representing occupied and vacant sites into a greyscale image using mat2gray. Henceforth, the procedure for analysing the experimental images and simulation data is identical. Edges are detected using edge with the Canny method [36] and a threshold between 0.04 and 0.1. Detected edges weaker than the threshold are ignored. Remaining edges are dilated, using imdilate, by a stretching element, defined using strel, with a square element of size seven. Any remaining vacant spaces are filled, using imfill, after which the dilation was reversed by eroding the image with the stretching element, defined previously, using imerode. The edges within the image were smoothed using medfilt2, and the area enclosed by the leading edge estimated using regionprops. For illustrative purposes, this algorithm was applied to the simulation data in figure 1*c* and the detected edge is superimposed in figure 1*d*. To estimate the vertical position of the leading edge, *Y*, we use2.2
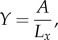
where *A* is the area enclosed by the detected leading edge. The average position of the leading edge, *Y*, is superimposed in figure 1*d*. To estimate how *Y* changes with time, we repeat the process at many time points and subtract the initial position to give a measure of the net displacement of the leading edge as a function of time. Schematic results in figure 1*h* indicate how the net displacement of the leading edge evolves with time for a representative set of experimental and averaged simulation data. We acknowledge that the edge detection could have been performed with ImageJ rather than Matlab. For this work, we chose to use Matlab because our previous comparison of Matlab and ImageJ edge detection algorithms showed that Matlab allows greater flexibility in the choice and control of threshold and dilation parameters [26].

## 3. Results

In this work, we will generate, and refer to, two distinct types of data: *experimental data* and *averaged simulation data*. The differences between these types of data can be described as follows:

— *Experimental data*. Describes the position of the leading edge as a function of time obtained from a single experiment. Furthermore, we consider two different ways of generating experimental data:
  (i) *In vitro experimental data*. Corresponds to data from experimental images.
  (ii) *In silico experimental data*. Corresponds to data from discrete simulation images.
— *Averaged simulation data*. Describes the average position of the leading edge, where the average has been constructed using data from many identically prepared realizations of the discrete model, that is, simulations performed with an identical algorithm, initial condition and parameters.

We construct the averaged simulation data using3.1
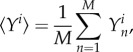
where 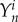 is the position of the leading edge, at time step *i*, in the *n*th identically prepared realization and *M* is the total number of identically prepared realizations. To measure the differences between different sets of experimental data and averaged simulation data, we define3.2
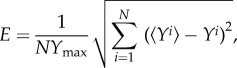
where 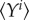 is the position of the leading edge, at time step *i*, using averaged simulation data, *Y^i^* is the position of the leading edge, at time step *i*, using experimental data, *N* is the number of time steps and *Y*_max_ is the maximum value of *Y^i^*, for *i* = 1, 2, 3, …, *N*.

### 3.1. Naive parameter recovery

**Figure 2.**
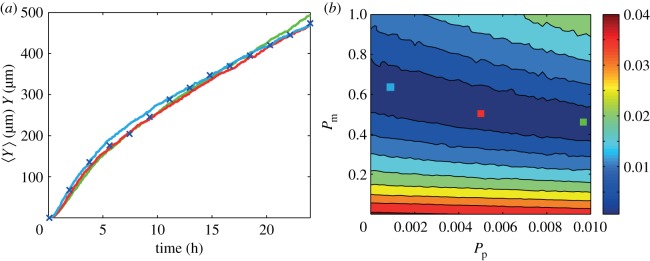
Comparison of *in silico* experimental data and averaged simulation data. (*a*) Leading edge *in silico* experimental data correspond to (*P*_m_, *P*_p_) = (0.5, 5 × 10*^−^*^3^) (blue crosses). Data are presented at every 20th time step. (*b*) Contour plot of *E* (equation (3.2)) measuring the difference between the *in silico* experimental data and averaged simulation data within the region *P*_m_ ∈ [0, 1], *P*_p_ ∈ [0, 0.01]. Simulation parameters are *M* = 10, *Y*_0_ = 750 µm, Δ = 25 µm and *τ* = 0.09191 h, with a final time of 24 h. The contour plot of *E* was generated by considering 2601 different parameter combinations; 51 equally spaced values of *P*_m_, and 51 equally spaced values of *P*_p_. The light blue, green and red coloured squares in (*b*) correspond to three different parameter combinations: (*P*_m_, *P*_p_) = (0.6, 1.4 × 10*^−^*^3^), (0.46, 8.8 × 10*^−^*^3^) and (0.5, 5 × 10*^−^*^3^), respectively. Averaged simulation data from these three different parameter combinations are superimposed in (*a*), showing that all three parameter combinations lead to indistinguishable short-time leading edge data. All averaged simulation data are insensitive to *τ*.

To explore whether it is possible to reliably estimate *D* and *λ* from short-time leading edge data, we first analyse a representative set of *in silico* experimental data corresponding to (*P*_m_, *P*_p_) = (0.5, 5 × 10*^−^*^3^), with Δ = 25 µm, which is reported in figure 2*a*. We note that it is difficult to draw specific conclusions by a simple visual inspection of this dataset, with the exception that it appears that the front speed is not constant over this time interval. To analyse this data, we generate a suite of averaged simulation data, sampling 2601 equally spaced parameter combinations within the region *P*_m_ ∈ [0, 1], *P*_p_ ∈ [0, 0.01], and we present a contour plot of *E*, given by equation (3.2), in figure 2*b*. We expect that if there is a unique choice of *D* and *λ* that matches the data in figure 2*a*, we would see a unique minimum on the *E* surface. Instead, we observe a relatively large, flat region, within which *E* takes on small, indistinguishable, values. This region extends right across this portion of the parameter space, indicating that there are many combinations of *D* and *λ* which match the experimental data equally well. To demonstrate that our observations for this parameter set hold more generally, we repeated the process focusing on *in silico* experimental data with a higher proliferation rate and found similar results (electronic supplementary material).

To demonstrate the redundancy in the short-time leading edge data, we choose three different combinations of (*P*_m_, *P*_p_), highlighted in figure 2*b*, and we superimpose the corresponding averaged leading edge data on the experimental data in figure 2*a*. Comparing these datasets confirms that there are several parameter combinations which give indistinguishable short-time leading edge data. Furthermore, we found that any parameter combination within the dark blue region in figure 2*b* also gives averaged simulation data that matches the experimental data (not shown). These results indicate that short-time leading edge information should be treated with care because a standard model calibration procedure may not provide useful information.

### 3.2. Parameter recovery accounting for the separation of time scales

Our results in §3.1 imply that additional information needs to be incorporated into our parameter estimation procedure if we are to infer useful information from short-time leading edge data. Here, we make use of the fact that there is a large separation of time scales between cell proliferation processes and cell motility processes. Typical estimates of the cell doubling time are approximately 15–30 h [3,19], whereas the time scale of cell motility events is approximately 10–20 min [37]. This separation of time scales implies that the first part of the leading edge time-series data will be dominated by the influence of cell motility, and we can make use of this information by dividing our time-series data into two intervals: (i) *t* < *T* and (ii) *t* > *T*, where *T* is a time interval during which the motion of the leading edge is dominated by cell motility. Intuitively, we expect that *T* ought to be chosen to be much less than the cell doubling time, and we will discuss this choice in §3.3.

To make use of this separation of time scales, we estimate *P*_m_ and *P*_p_ iteratively as follows:

— *Step 1.* Estimate *P*_m_ by considering experimental data for *t* < *T*, we set *P*_p_ = 0 and systematically vary *P*_m_ so that our averaged simulation data match the experimental data.
— *Step 2*. Estimate *P*_p_ by considering experimental data for *t* > *T*, we set *P*_m_ to be the value found previously, and we systematically vary *P*_p_ so that our averaged simulation data match the experimental data.
— *Step 3.* Re-estimate *P*_m_ by considering experimental data for *t* < *T*, we set *P*_p_ to be the value found in step 2, and we systematically vary *P*_m_ so that our averaged simulation data match the experimental data. Repeat steps 2 and 3 until both *P*_m_ and *P*_p_ converge.

**Figure 3.**
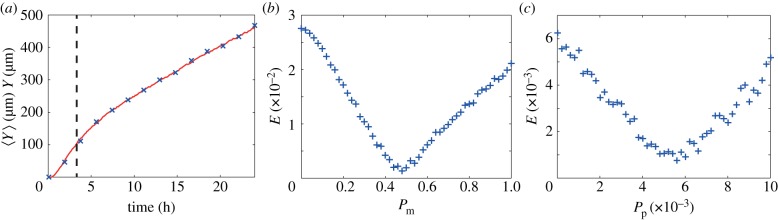
Parameter recovery for *in silico* experimental data using the iterative separation of time scales approach. (*a*) *In silico* experimental data, at every 20th time step, with (*P*_m_, *P*_p_) = (0.5, 5 × 10*^−^*^3^) (blue crosses). The vertical line represents *T* = 3 h. (*b*) Observing the minimum value of *E* (equation (3.2)) measuring the difference between the *in silico* experimental data and averaged simulation data for *t* < 3 h suggests that *P*_m_ is approximately 0.48. The averaged simulation data correspond to 51 equally spaced values of *P*_m_ in the interval *P*_m_ ∈ [0, 1], and *P*_p_ = 0. (*c*) *E* (equation (3.2)) measuring the difference between the *in silico* experimental data and averaged simulation data for 3 < *t* < 24 h. The averaged simulation data correspond to 51 equally spaced values of *P*_p_ in the interval *P*_p_ ∈ [0, 0.01] and *P*_m_ = 0.48, giving *P*_p_ ≈ 5.6 × 10*^−^*^3^. Simulation data were generated with *M* = 10, *Y*_0_ = 750 μm, Δ = 25 μm and *τ* = 0.09191 h, with a final time of 24 h. *P*_m_ and *P*_p_ required two iterations to converge. All averaged simulation data are insensitive to *τ*.

We now apply this method to *in silico* experimental data and then examine *in vitro* data in §3.3. Figure 3*a* shows same *in silico* experimental data presented previously in figure 2*a*. The results from estimating *P*_m_ using the iterative procedure are given in figure 3*b* and show that by choosing *T* = 3 h and focusing on the interval *P*_m_ ∈ [0, 1], we observe a relatively well-defined minimum in the plot of *E* indicating that we have *P*_m_ ≈ 0.48. The results from estimating *P*_p_ are given in figure 3*c* and show that with *T* = 3 h and *P*_m_ = 0.48, we observe a well-defined minimum in *E* indicating that we have *P*_p_ ≈ 5.6 × 10*^−^*^3^. We note that it took two iterations for *P*_m_ and *P*_p_ to converge. These parameter estimates are a great improvement on the results in §3.1 where we found it was impossible to distinguish between many different parameter combinations. We note that our parameter estimates do not precisely coincide with the expected values of *P*_m_ = 0.50 and *P*_p_ = 5 × 10*^−^*^3^, and there are two potential explanations for this. First, our *in silico* experimental data correspond to one realization of the discrete model which might not be representative of the expected behaviour we would observe when considering many identically prepared realizations. Second, when we generated the averaged simulation data, we only used a modest value of *M* = 10, and we expect that our estimate could be improved by increasing *M*. To further illustrate the robustness of our approach, we also applied it to data generated using several different parameter combinations, including some for larger proliferation rates, and we found that this method also gave reliable parameter estimates for these additional cases (electronic supplementary material).

Once we have obtained estimates of *P*_m_ and *P*_p_, it is possible to re-examine the suitability of our choice of *T*. Our estimate of *P*_p_ indicates that the average time taken for an isolated agent to undergo a proliferation event is approximately 18 h, whereas our estimate of *P*_m_ indicates that the average time taken for an isolated agent to undergo a motility event is approximately 30 min. These time scales give a physical explanation for why our choice of *T* = 3 h is sufficient, because agents have plenty of opportunity to undergo motility events during the first 3 h of the simulation, whereas there is hardly any opportunity for proliferation to occur during this interval. To further demonstrate the robustness of our results, we repeated the process of estimating *P*_p_ and *P*_m_ using the data in figure 3*a* and found that we obtained excellent estimates of the parameters regardless of whether we chose *T* = 2, 3 or 4 h.

### 3.3. *In vitro* data

**Figure 4.**
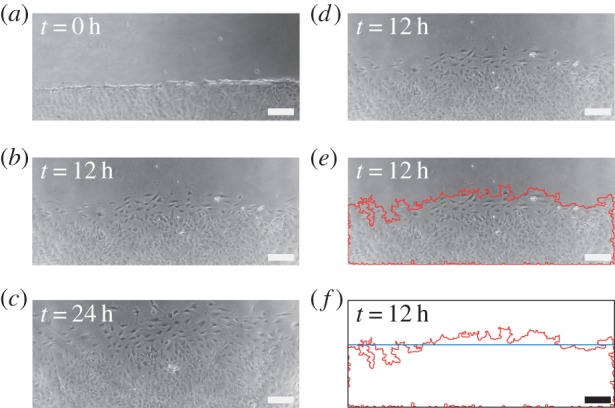
Time evolution of a scratch assay with 3T3 fibroblast cells. Experimental images are shown at: (*a*) 0, (*b*) 12 and (*c*) 24 h. To illustrate the application of the edge detection algorithm, we show in (*d*) the image at 12 h and in (*e*) we superimpose the detected leading edge (red). (*f*) Detected leading edge (red) and the average position of the front (blue). Scale bar corresponds to 200 µm.

**Figure 5.**
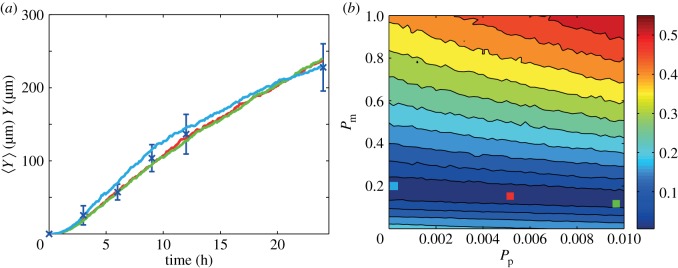
Comparison of *in vitro* experimental data and averaged simulation data. (*a*) Leading edge *in vitro* data (blue crosses) are presented with the error bars indicating 1 s.d. from the mean. (*b*) Contour plot of *E* (equation (3.2)) measuring the difference between the *in vitro* data and the averaged simulation data in the region *P*_m_ ∈ [0, 1], *P*_p_ ∈ [0, 0.01]. Simulation parameters are *M* = 10, *Y*_0_ = 750 µm, Δ = 25 µm and *τ* = 0.09191 h, with a final time of 24 h. The contour plot of *E* was generated by considering 2601 different parameter combinations; 51 equally spaced values of *P*_m_ and 51 equally spaced values of *P*_p_. The red, green and light blue coloured squares in (*b*) correspond to three different parameter combinations: (*P*_m_, *P*_p_) = (0.16, 5.4 × 10*^−^*^3^), (0.14, 9.6 × 10*^−^*^3^) and (0.2, 2 × 10*^−^*^4^), respectively. Averaged simulation data from these three different combinations are superimposed in (*a*), showing that all three parameter combinations lead to indistinguishable short-time leading edge data. All averaged simulation data are insensitive to *τ*.

We obtained *in vitro* experimental data for a scratch assay using 3T3 fibroblast cells as described in §2.1. At each time point, we took four different images, at different spatial locations, in the scratch assay. The field of view in each image is approximately 2 mm wide and 0.8 mm high. The spatial location of the four sets of images is approximately evenly spaced along the edge of the scratch within the red rectangle in figure 1*a*. One set of such images, at *t* = 0, 12 and 24 h, is presented in figure 4*a*–*c*. An example of the results from the edge detection algorithm, applied to the image at *t* = 12 h, is illustrated in figure 4*d*–*f*. Results summarizing the average position of the leading edge as a function of time are given in figure 5*a*, and the original dataset from the four sets of images at all time points is given in the electronic supplementary material.

We first apply the naive parameter recovery method, described in §3.1, where we consider the difference between our *in vitro* experimental data were averaged simulation data using the entire time series of leading edge data. The averaged simulation data were generated using 2601 equally spaced parameter combinations within the region *P*_m_ ∈ [0, 1], *P*_p_ ∈ [0, 0.01]. Results in figure 5*b* show a contour plot of *E*, defined by equation (3.2), which confirms that there is a large region within the parameter space for which the short-time leading edge data are indistinguishable. To confirm that multiple parameter combinations match the *in vitro* experimental data, we consider three distinct parameter pairs, highlighted in figure 5*b*, and superimpose the corresponding averaged simulation data in figure 5*a*.

**Figure 6.**
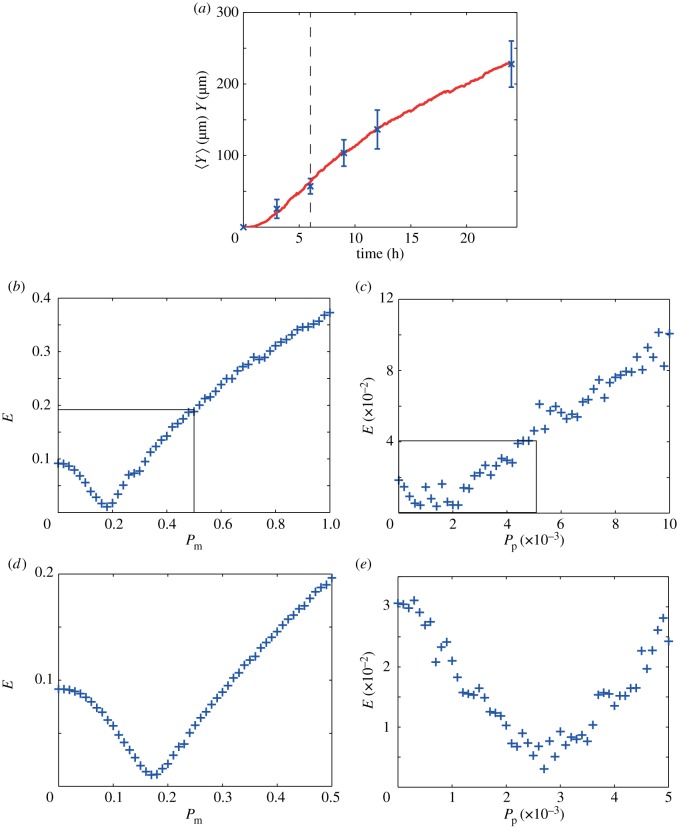
Parameter recovery for *in vitro* data using the separation of time scales approach. (*a*) *In vitro* data (blue crosses) showing the average position of the leading edge with the error bars denoting 1 s.d. from the mean (see the supplementary material for the original dataset). The vertical line indicates *T* = 6 h. (*b*) Plot of *E* (equation (3.2)) measuring the difference between the *in vitro* experimental data and averaged simulation data for *t* < *T* with *P*_p_ = 0. The plot of *E* was generated by considering 51 equally spaced values of *P*_m_ within the interval *P*_m_ ∈ [0, 1] and indicates that *P*_m_ is approximately 0.18. (*c*) Plot of *E* measuring the difference between the *in vitro* experimental data and the averaged simulation data for *t* > *T* with *P*_m_ = 0.18. The plot of *E*, generated using 51 equally spaced values of within the interval *P*_p_ ∈ [0, 0.01], indicates that the true value of *P*_p_ lies in the subinterval *P*_p_ ∈ [0, 5 × 10*^−^*^3^]. (*d*) Plot of *E* measuring the difference between the *in vitro* experimental data and the averaged simulation data for *t* < *T* with *P*_p_ = 0. The plot of *E*, generated using 51 equally spaced values of *P*_m_ within the subinterval *P*_m_ ∈ [0, 0.5], indicates that an improved estimate is *P*_m_ ≈ 0.17. (*e*) Plot of *E* measuring the difference between the *in vitro* experimental data and the averaged simulation data for *t* > *T* with *P*_m_ = 0.17. The plot of *E*, generated using 51 equally spaced values of *P*_p_ within the subinterval *P*_p_ ∈ [0, 5 × 10*^−^*^3^], indicates that the true value of *P*_p_ is approximately 2.7 × 10*^−^*^3^. Averaged simulation data showing the position of the leading edge with *P*_m_ = 0.17 and *P*_p_ = 2.7 × 10*^−^*^3^ are superimposed on the *in vitro* experimental data in (*a*). All simulation data correspond to *Y*_0_ = 750 µm, Δ = 25 µm and *τ* = 0.09191 h. Results in (*b*,*c*) correspond to *M* = 10, while results in (*d*,*e*) correspond to *M* = 50. Our estimates of *P*_m_ and *P*_p_ required one iteration to converge. All averaged simulation data are insensitive to *τ*.

We now apply the approach described in §3.2 to our *in vitro* data choosing *T* = 6 h. Results in figure 6*a* show the averaged experimental data. A plot of *E*, given in figure 6*b*, constructed using 51 equally spaced values of *P*_m_ in the interval *P*_m_ ∈ [0, 1], with *P*_p_ = 0, indicates that the optimal value of *P*_m_ is approximately 0.18. A plot of *E*, given in figure 6*c*, constructed using 51 equally spaced values of *P*_p_ in the interval *P*_p_ ∈ [0, 0.01], with *P*_m_ = 0.18, indicates that the proliferation parameter lies within the subinterval *P*_p_ ∈ [0, 5 × 10*^−^*^3^]. We now refine our parameter estimates by repeating the process and increasing the number of realizations used to generate the averaged simulation data from *M* = 10 to *M* = 50. Furthermore, we now focus our attention on the subintervals *P*_m_ ∈ [0, 0.5] and *P*_p_ ∈ [0, 5 × 10*^−^*^3^], highlighted by the rectangles superimposed on figure 6*b*,*c*. By repeating the parameter estimation process, we obtained the refined results shown in figure 6*e*,*f*, indicating that the optimal parameter pair is (*P*_m_, *P*_p_) = (0.17, 2.7 × 10*^−^*^3^), or (*D*, *λ*) ≈ (300 µm^2^ h^−1^, 0.03 h^−1^). To quantify the uncertainty in our estimates, we repeated the same process using the mean experimental data ±1 sample standard deviation of the experimental data. This gave *P*_m_ = 0.17 (0.14−0.20) and *P*_p_ = 2.7 × 10*^−^*^3^ (1.6 × 10*^−^*^3^ − 3.5 × 10*^−^*^3^), where the ranges in the parentheses indicate an estimate of the uncertainty. Our estimates of *P*_m_ and *P*_p_ were obtained using just one iteration.

To explore whether our results are sensitive to our choice of *T*, we repeated the process using *T* = 9 h and found that this also gave (*P*_m_, *P*_p_) = (0.17, 2.7 × 10*^−^*^3^), or (*D*, *λ*) ≈ (300 µm^2^ h^−1^, 0.03 h^−1^), indicating that our results are relatively insensitive to *T*. The reason for this insensitivity can be explained by considering the time scales implied by our parameter estimates. Our estimate of *λ* indicates that the average time required for an isolated cell to proliferate is approximately 34 h. In comparison, our estimate of *D* indicates that the average time taken for an isolated cell to undergo a motility event is approximately 30 min. This indicates that that either *T* = 6 or 9 h is appropriate because either of these time scales is sufficiently small relative to the proliferation time scale is well as being sufficiently large compared with the motility time scale. Averaged simulation data produced using (*P*_m_, *P*_p_) = (0.17, 2.7 × 10*^−^*^3^) are superimposed in figure 6*a*, confirming that the simulated leading edge data match the measurements. We note that our parameter estimates are consistent with Tremel *et al*.'s [22] previously reported estimates. However, we would also like to point out that our estimates of *D* and *λ* were obtained simply and inexpensively, using only short-time leading edge data, whereas Tremel's results were obtained by constructing cell density profiles and tracking individual cells, both of which are time consuming and expensive.

## 4. Discussion and conclusion

Moving cell fronts [7–10] play a key role in development, disease and tissue repair. The rate at which the cell front moves depends both on the motility and proliferation of individual cells within the population. Mathematical models can be used to interpret scratch assays, with some previous studies focusing exclusively on matching experimental estimates of the front speed with the long-time asymptotic wave speed of the travelling wave solution of the Fisher–Kolmogorov equation, 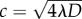 [14]. This approach suffers from two limitations. First, travelling wave solutions require a large amount of time to develop, whereas most scratch assays are performed for short-time intervals. Second, even if large-time experimental data are available, this approach determines the product, *λD*, and not the values of *λ* and *D* separately [19,20]. Other methods for interpreting scratch assays have involved calibrating the numerical solution of a reaction–diffusion equation to observed cell density profiles [3,21,22] to provide estimates of *λ* and *D*. Unfortunately, this approach is expensive and time consuming since it requires either a direct or indirect method for counting individual cells to construct the cell density profiles.

In this work, we describe a different approach for analysing scratch assays relying only on determining short-time leading edge data. Our method can be implemented either for new experimental images or, retrospectively, using previously published images. The simplicity of our approach derives from the fact that we do not require any analysis or counting of individual cells. Using a discrete model of cell motility and cell proliferation, we show that care ought to be exercised when analysing short-time leading edge data because a straightforward model calibration procedure, whereby we match the entire time history of the position of the leading edge, reveals that there are many parameter combinations for which the short-time leading edge data from the model are equivalent. To overcome this, we make use of the fact that cell migration takes place on a short time scale compared with cell proliferation, and we introduce a new iterative method where we analyse the leading edge time-series data in two steps. First, we analyse the interval *t* < *T*, setting *P*_p_ = 0 in the model, to provide an estimate of *P*_m_. Second, we analyse the time interval *t* > *T*, using our previously determined estimate of *P*_m_, to provide an estimate of *P*_p_. These two steps can be applied iteratively until our estimates converge to within some tolerance. Our approach relies on estimating some time, *T*, which is sufficiently large compared with the time scale of cell migration, yet is sufficiently small compared with the time scale of proliferation. We confirm our approach using both *in silico* and *in vitro* data, and we note that our estimates of *D* and *λ* for the *in vitro* data are consistent with previously published values for the same cell line in a similar experiment [22].

As we demonstrate, once the data have been analysed to produce an estimate of *D* and *λ*, we can test the sensitivity of our estimates to our choice of *T*. For our *in silico* data, we found that we obtained similar results regardless of whether we chose *T* = 2, 3 or 4 h. Similarly, for our *in vitro* data, where we had less experimental data points from which to choose *T*, we found that we obtained the same values for *D* and *λ* regardless of whether we chose *T* = 6 or 9 h.

Our parameter estimates for the *in vitro* data indicate that care should be taken when interpreting leading edge data with the long-time asymptotic wave speed expression for the Fisher–Kolmogorov equation [18–20]. Our parameter estimates for the *in vitro* data correspond to (*D*, *λ*) ≈ (300 µm^2^ h^−1^, 0.03 h^−1^). While it is possible to use these parameters to estimate the speed of the travelling wave solution of the Fisher–Kolmogorov equation [18–20], this result is valid only in the long-time limit, *t* → *∞*. As our experimental results have been reported over a time interval which is less than the doubling time, we expect that it is inappropriate to use such a result because there has been insufficient time for the travelling wave to form. Indeed, comparing the slope of the data in figure 5*a* with 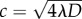, evaluated using our parameter estimates, confirms that these approaches give different estimates of the front speed.

Our approach of combining simulation data with automated leading edge analysis can be extended in several ways. One important point, not considered here, is that certain cells, such as melanoma [33] and glioma cells [37], exhibit significant cell-to-cell adhesion. To incorporate cell-to-cell adhesion, we could consider a different discrete model with an additional parameter controlling the adhesion strength [37]. Under these conditions, it would be interesting to explore whether the three parameters governing cell migration, cell proliferation and the strength of adhesion could be uniquely determined by short-time leading edge data. Alternatively, we could apply our model to scratch assays performed on different substrates [19,20] to analyse the effect of cell-to-substrate adhesion. Another approach may be to apply our model to narrow wounds, where cell proliferation is negligible or absent [38]. A further application of our model would be to analyse a series of scratch assays where we considered some control assay relative to a set of other assays where a chemical inhibitor or promotor has been applied. Our approach could be used to determine precisely how *D* and/or *λ* varies as a function of the concentration of the chemical, and therefore play a role in the design of intervention strategies aimed at manipulating the movement of cell fronts.

## Supplementary Material

Supplementary Material
