# Supplementary material for: How much information can be obtained from tracking the position of the leading edge in a scratch assay?
Source: J R Soc Interface. 2014 Aug 6;11(97):20140325. doi: 10.1098/rsif.2014.0325 (PMC4208362; doi:10.1098/rsif.2014.0325)
Supplement: Supplementary Material [file rsif20140325supp1.pdf]

# How much information can be obtained from tracking the position of the leading edge in a scratch assay?

Stuart T Johnston[1,2] *Matthew J Simpson[1,2] DL Sean McElwain[1,2]

**Keywords**: cell motility; cell proliferation; scratch assay; edge detection; cancer.

1. Mathematical Sciences, Queensland University of Technology, Brisbane, Australia. * matthew.simpson@qut.edu.au.

2. Institute of Health and Biomedical Innovation, Queensland University of Technology, Brisbane, Australia.

## 1 Supplementary Data

### 1.1 Reaction-diffusion model

The continuum-limit description of the discrete model described in Section 2.2 is a two-dimensional generalization of the Fisher-Kolmogorov equation [13],

$$\frac{\partial u}{\partial t} = D\nabla^2 u + \lambda u(1-u),$$

where $0 \leq u \leq 1$ is a scaled density, $D$ is the diffusivity and $\lambda$ is the proliferation rate. This model, applied in one-dimensional geometry, where $\nabla^2 u = \partial^2 u/\partial x^2$, supports travelling wave solutions in the long time limit, $t \to \infty$ [14]. For initial conditions with compact support the long time travelling wave speed is $c = \sqrt{4D\lambda}$ [14].

### 1.2 Naïve Parameter Recovery with High Proliferation Rate

We now consider results that are equivalent to those presented in Section 3.1 (main paper) except that we consider a higher proliferation rate with parameters $(P_m, P_p) = (0.8, 0.02)$. The experimental data is given in Figure 1(a) and the corresponding contour plot of $E$, generated using Equation (4) (main paper), is given in Figure 1(b) indicates that there is no single well-defined minimum on this surface. Instead, we observer a large dark blue region, extending right across the parameter space, from which any combination of $D$ and $\lambda$ produce indistinguishable short time leading edge data. We demonstrate this by choosing three different parameter combinations, highlighted in Figure 1(b), and give the corresponding averaged simulation data in Figure 1(a).

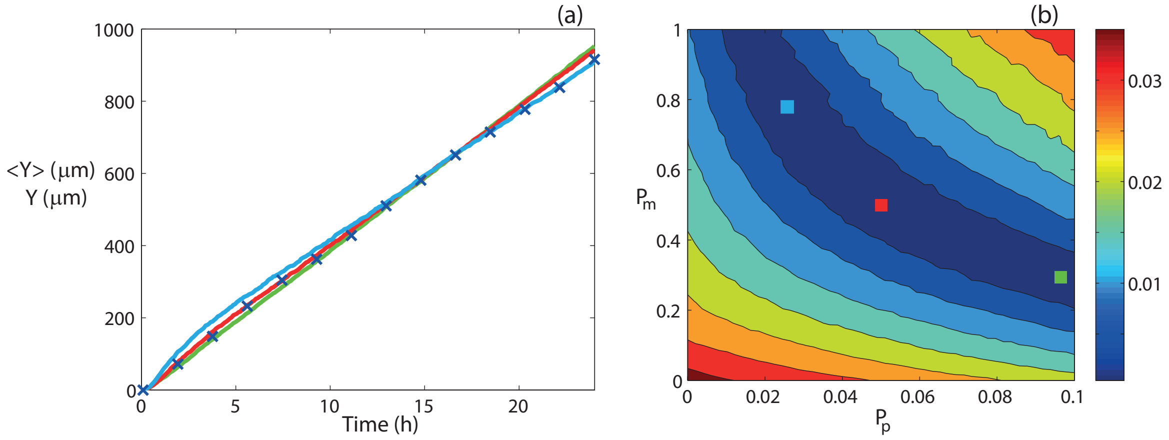


Figure 1: Comparison of *in silico* experimental data and averaged simulation data. (a) Leading edge *in silico* experimental data (blue crosses) corresponds to $(P_m, P_p) = (0.5, 0.02)$ (blue crosses). Data is presented every $20^{\text{th}}$ time step. (b) Contour plot of $E$ (Equation (4), main paper) measuring the difference between the *in silico* data and averaged simulation data within the region $P_m \in [0, 1], P_p \in [0, 0.1]$. Simulation parameters are $M = 10, Y_0 = 750$ $\mu$m, $\Delta = 25$ $\mu$m and $\tau = 0.09191$ h, with a final time of 24 h. The contour plot of $E$ was generated by considering 2601 different parameter combinations; 51 equally-spaced values of $P_m$ and 51 equally-spaced values of $P_p$. The red, green and light blue coloured squares in (b) correspond to three different parameter combinations: $(P_m, P_p) = (0.5, 0.05), (0.32, 0.096)$ and $(0.78, 0.028)$, respectively. Averaged simulation data from these three different parameter combinations are superimposed in (a), showing that all three parameter combinations lead to indistinguishable short time leading edge data. All averaged simulation data are insensitive to $\tau$.

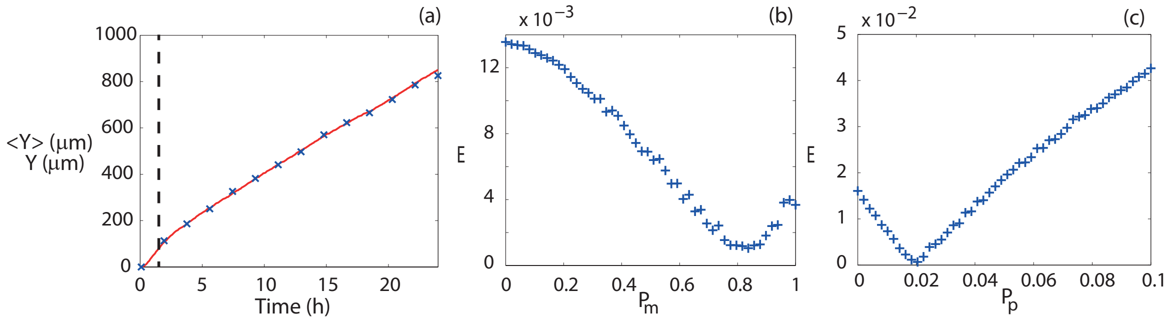


Figure 2: Recovery of parameter values from *in silico* experimental data using a separation of timescales. (a) *In silico* experimental data (blue crosses) with $(P_m, P_p) = (0.8, 0.02)$. The vertical line denotes $T = 1.5$ h. (b) Shows a plot of $E$ (Equation 4, main paper) measuring the difference between the *in silico* experimental data and averaged simulation data for $t < T$ with $P_p = 0$. The plot of $E$ was generated by considering 51 equally-spaced values of $P_m$ within the interval $P_m \in [0, 1]$, and indicates that $P_m$ is approximately 0.84. (c) Plot of $E$ measuring the difference between the *in silico* experimental data and averaged simulation data for $t > T$ with $P_m = 0.84$. The plot of $E$, generated using 51 equally-spaced values of $P_p$ within the interval $P_p \in [0, 0.1]$, indicates that $P_p$ is approximately 0.022. All simulations were performed with $M = 10, Y_0 = 750$ $\mu$m, $\Delta = 25$ $\mu$m and $\tau = 0.09191$ h, with a final time of 24h. $P_m$ and $P_p$ took three iterations to converge. All simulation results are independent of the duration of the time step.

### 1.3 Parameter Recovery Accounting for the Separation of Time Scales with High Proliferation Rate

We now apply the technique described in Section 3.2 (main paper) to the *in silico* experimental data from Figure 1(a) with $T = 1.5$ h, as shown in Figure 2(a). We consider the leading edge data from the first 1.5 h of the experiment and scan the parameter space in the interval $P_m \in [0, 1]$. Results in Figure 2(b) indicate that we have $P_m \approx 0.84$. We then consider the leading edge data in the interval $1.5 < t < 24$ h and scan the parameter space in the interval $P_p \in [0, 0.1]$. Results in Figure 2c indicate that we have $P_p \approx 0.022$, which provides an excellent match to the real parameters. Our procedure to estimate $P_m$ and $P_p$ required three iterations to converge.

### Experimental data

The experimental data set describing $n = 4$ images from the scratch assay, performed according to the methods outlined in Section 2.1, are presented in Table 1.

Table 1: Data describing the mean, $\langle Y \rangle$, and standard deviation, $\sigma$, of the leading edge position from four images of the scratch assay. Data corresponds to $t = 0, 3, 6, 9, 12$ and 24 h, as indicated.

| | $t = 0$ h | $t = 3$ h | $t = 6$ h | $t = 9$ h | $t = 12$ h | $t = 24$ h |
|---|---|---|---|---|---|---|
| $Y_1$ ($\mu$m) | 0 | 39.99 | 46.38 | 109.53 | 161.82 | 183.84 |
| $Y_2$ ($\mu$m) | 0 | 13.87 | 52.46 | 82.18 | 130.29 | 230.53 |
| $Y_3$ ($\mu$m) | 0 | 14.85 | 71.74 | 97.34 | 125.65 | 261.14 |
| $Y_4$ ($\mu$m) | 0 | 33.04 | 58.27 | 125.60 | 155.06 | 235.92 |
| $\langle Y \rangle$ ($\mu$m) | 0 | 25.44 | 57.21 | 103.66 | 136.45 | 227.86 |
| $\sigma$ ($\mu$m) | 0 | 13.11 | 31.03 | 18.42 | 27.12 | 32.34 |
